# A Novel Counterfeit Feature Extraction Technique for Exposing Face-Swap Images Based on Deep Learning and Error Level Analysis

**DOI:** 10.3390/e22020249

**Published:** 2020-02-21

**Authors:** Weiguo Zhang, Chenggang Zhao, Yuxing Li

**Affiliations:** 1 College of Computer Science and Technology, Xi’an University of Science and Technology, Xi’an 710054, China; wgzhang@scut.edu.cn; 2 School of Automation and Information Engineering, Xi’an University of Technology, Xi’an 710048, China

**Keywords:** deep learning, feature extraction, DeepFake detection, ELA detection, CNN

## Abstract

The quality and efficiency of generating face-swap images have been markedly strengthened by deep learning. For instance, the face-swap manipulations by DeepFake are so real that it is tricky to distinguish authenticity through automatic or manual detection. To augment the efficiency of distinguishing face-swap images generated by DeepFake from real facial ones, a novel counterfeit feature extraction technique was developed based on deep learning and error level analysis (ELA). It is related to entropy and information theory such as cross-entropy loss function in the final softmax layer. The DeepFake algorithm is only able to generate limited resolutions. Therefore, this algorithm results in two different image compression ratios between the fake face area as the foreground and the original area as the background, which would leave distinctive counterfeit traces. Through the ELA method, we can detect whether there are different image compression ratios. Convolution neural network (CNN), one of the representative technologies of deep learning, can extract the counterfeit feature and detect whether images are fake. Experiments show that the training efficiency of the CNN model can be significantly improved by the ELA method. In addition, the proposed technique can accurately extract the counterfeit feature, and therefore achieves outperformance in simplicity and efficiency compared with direct detection methods. Specifically, without loss of accuracy, the amount of computation can be significantly reduced (where the required floating-point computing power is reduced by more than 90%).

## 1. Introduction

Nowadays, with the popularization of smartphones and various face-swap applications, the manipulation of visual content is becoming increasingly common, which has been one of the most heated issues in the digital society. The core problem, more importantly, lies in the new generation of generative deep neural networks [1], which are capable of synthesizing videos from a large volume of training data with minimum manual editing. Furthermore, the emergence of DeepFake [2] greatly reduces the threshold of face forgery techniques. To be specific, DeepFake replaces the face in an original video with the face of another person using generative adversarial networks (GANs) [3]. As the GAN models were trained with tens of thousands of images, it is more likely to generate realistic faces that can be spliced into the original video more precisely. Through suitable post-processing, the video can achieve higher authenticity. In addition, with the advancement of deep learning, face-swap technology has been applied in numerous scenes including privacy protection, video synthesis as well as other innovative applications. Utilizing DeepFake or other technologies, one GPU and a large number of training data are only needed to produce a highly advanced face-swap video. Some technology enthusiasts have released quantities of live-performance videos altering ordinary people’s faces with the synthesized stars’ faces on the short video platform, ending up as a focus to be discussed. 

This technology has indeed aroused related concerns. Prior to this technology, it was generally believed that videos were reliable, even working as video evidence in multimedia forensics. However, the prevalence of DeepFake has made people realize that, in addition to short message service (SMS) and telephone fraud, people can even be defrauded by videos [4]. DeepFake technology has posed a challenge to the public trust level in the information age. Some even argue that this technology might hinder the development of society. 

The detection of such fake videos, as a result, has become extremely urgent. More researchers have begun to probe into detection methods, and seek to explore differences between real videos and fake ones. Two main methods—traditional methods and deep learning methods—have mainly been used. Further living body recognition in face recognition and multimedia forensics also provide some solutions. It is believed that deep learning can now generate realistic fake faces, can also detect and explore invisible forgery traces, and is able to identify forgery videos. Unlike traditional image forensic methods, deep learning methods integrate feature extraction and feature classification into a network structure, and realize an end-to-end effective algorithm of automatic feature learning classification. On the other hand, the traditional image forensic methods are usually not suitable for video, because compression will seriously reduce the quality of the data. As deep learning performs well in digital forensics, it disturbs the traditional image forensic methods.

For example, Pavel et al. [5] showed that the state-of-the-art face recognition methods based on visual geometry group (VGG) and Facenet neural networks were susceptible to DeepFake videos with 85.62% and 95.00% false acceptance rates (on high quality versions), respectively. This means that methods for detecting DeepFake videos are needed. Furthermore, their experiments revealed the complexity of detecting GAN-generated DeepFake videos with both face recognition and existing detection methods. Therefore, the further development of face-swap technology will make it even more so. Even worse, people will suffer from further application of the technology in illegal activities.

From the current research work, the application of deep learning in image forensics can be roughly divided into three levels, as shown in Table 1. The first level is simple migration, where the CNN structure commonly used in the field of computer vision is directly introduced into the field of image forensics. Li et al. [6] described a new deep learning based method that could effectively distinguish AI-generated fake videos from the real ones. They trained four CNN models: VGG16 [7], ResNet50, ResNet101, and ResNet152 [8]. The second level is to try to modify the network input. The original intention of such an attempt is driven by the essential difference between forensics and computer vision. Darius et al. [9] trained a convolution neural network to directly classify real faces and fake faces generated by DeepFake and Face2face [10]. They proposed detecting the forged videos of faces at a mesoscopic level of analysis. The third level is to modify the network structure, combining the actual problems of forensics to propose a network structure suitable for forensics. Among them, there are some detection methods of DeepFakes that are dependent on the inherent artifacts or inconsistencies. Li et al. [11] observed that DeepFake faces a lack of real eye blinking due to the scarcity of photos of closed eyes. Therefore, the lack of eye blinking can expose DeepFake videos with a CNN/RNN model. However, this method can be invalid by deliberately adding images with eyes closed in training. Li et al. [12] used the color differences between GAN generated images and real images in a non-RGB color space to classify them. Xin et al. [13] proposed a new method to detect and identify video forgery based on the inconsistency of the head positions. Peng et al. [14] proposed a two-stream network for face tamper detection. GoogleNet is trained to detect tampering artifacts in the face classification stream, and a patch on the basis of the triplet network is trained to leverage features capturing partial noise residuals and camera characteristics as the second stream. Despite the promising performance shown by this method, the methods above have drawbacks. Extracting features directly from the original image requires going through an extra series of training cycles, leading to lower efficiency.

Cpatel et al. [15] proposed a method to identify the forgery region frames from a given input video by using EXIF image tag information and ELA. Jeronymo et al. [16] presented a method for the detection of image forgery in lossy compressed digital images by using ELA and its noisy components were filtered with automatic wavelet soft-thresholding. Sudiatmika et al. [17] proposed a new system from a combination ELA and CNN to solve the problem of distinguishing real images from forged ones.

To resolve these problems, we proposed a novel counterfeit feature extraction technique to expose face-swap images based on the deep learning and ELA, which can effectively distinguish facial images generated by deep learning. Not only does the proposed technique retain the merits of existing techniques, but overcomes the demerits using ELA and our model, compared with other methods.

To summarize, our paper makes the following contributions: (1) a method of generating a face-swap image dataset by simulating the process of generating faces in DeepFake; and (2) a novel counterfeit feature extraction technique based on deep learning and ELA.

The rest of this paper is organized as follows. Section 2 presents the outline of our proposed method. Section 3 introduces the main methods such as dataset preprocessing, ELA processing. and our proposed CNN architectures. In Section 4, we train and evaluate our model on the Milborrow University of Cape Town (MUCT) database with negative face images, and compare it with other methods. Conclusions are drawn in Section 5.

## 2. Outline of the Proposed Method

**Figure 1:**
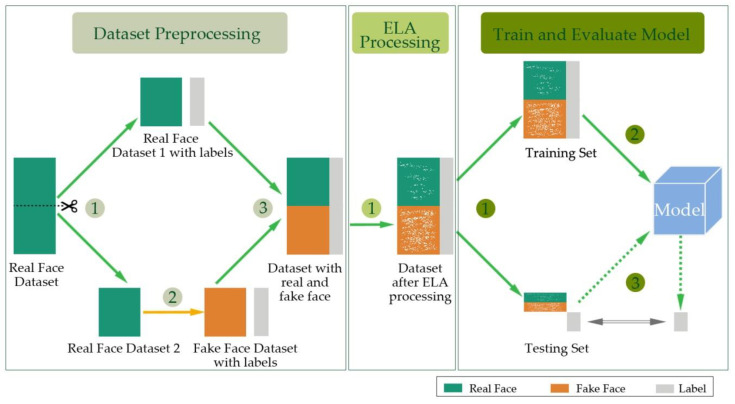
Outline of the proposed method.

In this section, we describe our method of detecting facial image counterfeits. Figure 1 outlines the proposed method.

Stage 1: Dataset Preprocessing.

(1) Divide the dataset (as marked with drakcyan blocks) that has only real faces into two parts randomly.

(2) One part generates fake faces (as marked with orange blocks), and attach the corresponding labels (as marked with gray blocks).

(3) The remaining part and the generated fake faces form a new dataset.

Stage 2: ELA Processing.

(1) Process the new dataset with the ELA method and form a new dataset (as marked with gray spot blocks).

Stage 3: Train and Evaluate Model. A binary classifier is trained by a convolutional neural network to distinguish whether the image is fake.

(1) Split the new dataset into training and testing subsets according to the proportion of 4:1.

(2) Train a model on the training set.

(3) Make predictions on the testing set, and compare the predicted and true labels.

## 3. Methods

### 3.1. Dataset Preprocessing

**Figure 2:**
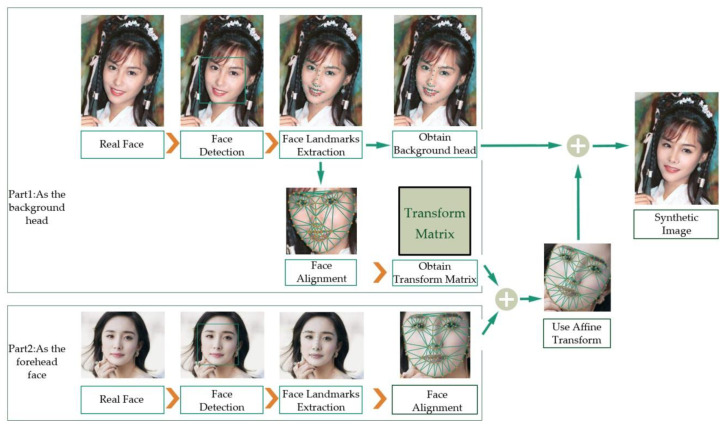
Overview of the DeepFake principle.

We analyzed the process of generating fake faces by DeepFake. The principle of DeepFake is shown as Figure 2. The core idea of DeepFake is the parallel training of two automatic encoders. Its principle can be summarized as follows: a neural network is trained to restore the distorted face of A to the original face by supervised learning, and the network is expected to have the ability to restore any face to the face of A.

As is shown in Figure 2, we can see the source image in the first rectangle, which is marked “Part 1” and will be the background head of the ultimate generated image. After a series of processing such as face detection, face landmark detection, and face alignment, its transformation matrix can be calculated and obtained. The target image is in the second rectangle, which is marked “Part 2” and will be transformed into the foreground face of the ultimate generated image. The target image goes through the same processing. According to the transformation matrix of the original image, the target image can be wrapped and transformed to obtain the synthetic forged image. After some post-processing such as applying boundary smoothing to compose the image, the ultimate synthetic image is generated.

Due to the limitations of computing resources and production time, the DeepFake algorithm can only generate limited resolutions and then perform affine transformation on those generated images such as scaling, rotation, and shearing to match and cover the original face that they will replace. This will result in two different image compression ratios between the fake face area as the foreground and the original area as the background, which would leave obvious counterfeit traces.

Our purpose here was to detect the artifacts introduced by the affine transformation steps in DeepFake production. In addition, since DeepFake needs to be trained for each pair of videos, which is time-consuming and resource-demanding, we did not use the DeepFake algorithm to create negative examples. Instead, we simplified the process of generating negative examples by simulating the process of generating faces in DeepFake.

**Figure 3:**
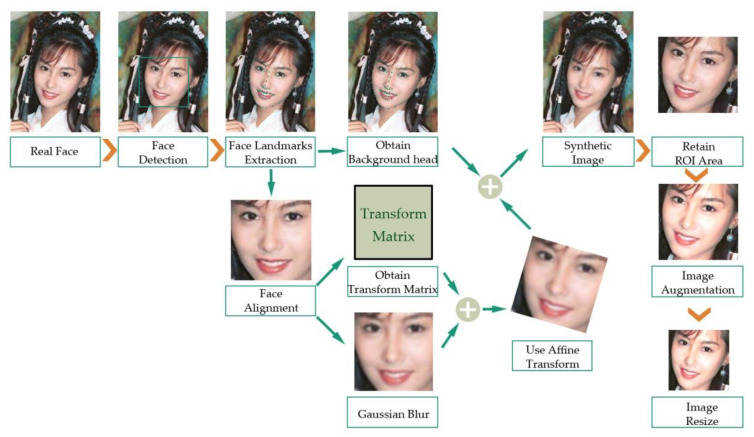
The flow chart of generating negative examples.

Specifically, we took the following steps to generate negative examples, as shown in Figure 3. First, we detected faces in the original images, and extracted face landmarks from each detected facial area. Then, according to the landmarks, we aligned the face and calculated the transformation matrix. Facial landmarks contain the location of important facial structural information such as the contours of the eyes, nose, and mouth. The different facial orientation in the images will interfere with the facial analysis. Therefore, we needed to align the facial area to the unified coordinate space according to the detected facial landmarks. That is to say, we adjusted the face of a possible side face or the face with an angle to the standard forward face, so that the aligned face was in the center of the image, the eyes were on the horizontal line and zoomed to a similar size.

The transformation matrix is acquired by face alignment, which in essence is to find the affine transformation matrix from shape A to shape B by using the least square method. For the two shape matrices with face landmarks, they can be marked with p and q, respectively. Each row of the matrix represents the x and y coordinates of a facial feature point. The face has 68 landmarks, so p and q can be marked as follows:

(1) $$p\in R^{68*2},\;q\in R^{68*2}$$

The above problem can be described as: two matrices p and q are known, and q can be obtained by the transformation:  q=sRp+T. Our goal was to calculate the zoom scale s, rotation angle θ, and translation displacement t when ∑∥sRp+T−q∥ is minimized. Thus, the process of solving the affine matrix can be written in the following mathematical form:

(2) $$argmin_{s,\theta ,t}\sum_{i=1}^{68}{∥sRp_{i}^{T}+T-q_{i}^{T}∥}^{2}$$

where $p_{i}$ is the row i of the p matrix; R is an orthogonal matrix; and the superscript T represents the transposition of the matrix. The matrix form is as follows:

(3) $$argmin_{s,R,T}∥sRp^{T}+T-q^{T}{∥}^{2}$$

where ∥ ∥F represents the Frobenius norm, which is the sum of squares of each term.

After the previous steps, we applied Gaussian blur to the adjusted face. According to the inverse of the transform matrix, the face was wrapped back to the original angle and covered the original face image.

In addition, we also needed to preprocess the images before training. Since the input of the convolution neural network is 128 × 128, the image area is limited. Therefore, it is important to retain the most effective and prominent signs of the forged area as our region of interest (ROI). We determined the ROI area according to the face landmarks. For the convenience of description in this paper, the rectangular region composed of the convex hull of facial landmarks (except the contour of the cheek) is called the minimum circumscribed rectangle.

Analyzed as above, the affine transformation mainly affects the inner region of the minimum circumscribed rectangle. As a result, there is an obvious contrast between the inner region and the outer adjacent region of the rectangle. There might be some forgery marks in vision. Therefore, we chose to retain the slightly larger rectangular area composed of the minimum circumscribed rectangle and its surrounding area as our ROI area, and removed the rest of the image last. Specifically, for all positive and negative examples in the dataset, only the above ROI area was preserved, which was slightly larger than the minimum circumscribed rectangle. 

**Figure 4:**
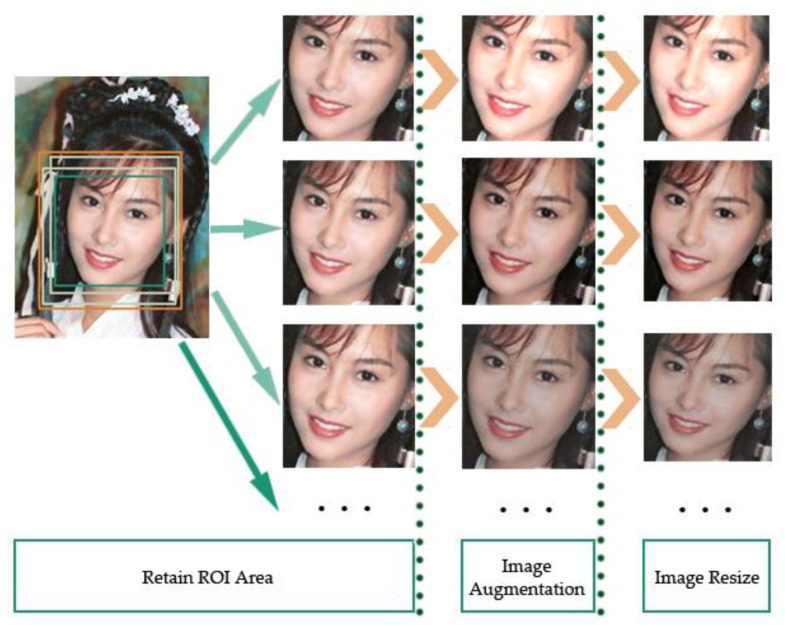
Some samples of the region of interest (ROI) area and processing results.

The two rectangle regions are represented as follows:

(4) $$\left[\begin{array}{ccc} \left(x_{1},y_{1}\right) & \cdots & \cdots \\ \vdots & \cdots & \vdots \\ \cdots & \cdots & \left(x_{2},y_{2}\right) \end{array}\right]\to \left[\begin{array}{ccc} \left(x\prime_{1},y\prime_{1}\right) & \cdots & \cdots \\ \vdots & \cdots & \vdots \\ \cdots & \cdots & \left(x\prime_{2},y\prime_{2}\right) \end{array}\right]$$

wherein the left matrix represents the minimum circumscribed rectangle that can cover all the facial landmarks (except the contour of the cheek), and corresponds to the green rectangle in the left image of Figure 4. $\left(x_{1},y_{1}\right)$ and $\left(x_{2},y_{2}\right)$ represent the coordinates of two diagonal vertices of the minimum circumscribed rectangle region. Its width and height are expressed as *h* and *w*, respectively. The right matrix represents the ROI rectangular region that we want to retain. $\left(x\prime_{1},y\prime_{1}\right)$ and $\left(x\prime_{2},y\prime_{2}\right)$ represent the coordinates of two diagonal vertices of the ROI rectangle region. This is slightly larger than the minimum circumscribed rectangle, and corresponds to the light yellow rectangle in the left image of Figure 3.

The conversion relationship between the two rectangular regions is illustrated as follows:

(5) $$x_{1}^{\prime }=\max\left\{0,x_{1}-delta\_x\right\}\;y_{1}^{\prime }=\max\left\{0,y_{1}-delta\_y\right\}\;x_{2}^{\prime }=\min\left\{w-1,\;x_{2}+delta\_x\right\}\;y_{2}^{\prime }=\min\left\{h-1,y_{2}+delta\_y\right\}$$

wherein the variables delta_x and delta_y is a random value [0, *h*/5] and [0, *w*/8].

(6) $$delta_{x}=rand\left\{0,\left(x_{2}-x_{1}\right)/8\right\}\;delta\_y=rand\left\{0,\left(y_{2}-y_{1}\right)/5\right\}$$

After selecting the ROI area, we removed the rest of the image. In order to simulate more different resolutions of face affine transform in reality, we aligned the faces into multiple scales and randomly selected one scale to enlarge the training diversity, as can be seen in Figure 4. The green rectangle represents the minimum circumscribed rectangle that can cover all the facial landmarks (except the contour of the cheek). The light yellow rectangle represents one of the ROI rectangular regions that we wanted to retain. The orange rectangle represents the maximal circumscribed rectangle that may be retained. The images in the second column in Figure 4 show some different ROI results.

We also used image augmentation technology to simulate different post-processing technologies that may exist in the DeepFake process, as can be seen in the third column of Figure 4. Specifically, for all images in the training dataset, we used image augmentation, which mainly includes shape transformation (such as rotation, scaling, flipping, translation, etc.) and color jittering (such as brightness, contrast, color distort, sharpness, etc.). We selected random values to match different effects, so that the images were slightly different for each epoch, increasing the diversity of the training dataset. Our approach also further dealt with the shape of the face area affine transformation to cope with the different post-processing techniques. After all of the above work, the image was resized to 128 × 128 for the next ELA processing.

### 3.2. The Error Level Analysis Processing

The error level analysis (ELA) method [18] is one of the techniques for detecting an image that has been tampered. The ELA method can obtain the compression distortion during lossy image compression. This method detects tampered images by storing images at a specific level of quality and calculating the ratio between compression levels. The local minimum in the image difference represents original regions, and the local maximum represents tampered regions. Typically, this method is performed on images with lossy compression formats such as JPEG.

Images will go through independent “lossy compression” in units of 8 × 8 pixels while being saved in JPEG format. Then, there are significant differences between the ELA of the original area and that of the spliced or modified one. If the image is modified, the compression difference of each 8 × 8 pixel region is not similar. We then check the “compression feature” of the tested image with an 8 × 8 pixel grid. Therefore, if the image is saved as a whole, the compression feature of the adjacent grid should be an approximately high-frequency white distribution. Instead, if it is saved after editing or modification, the ELA distribution between the grids will have obviously different characteristics, which is shown as a discontinuous high-frequency white distribution. The more times the images are stored or edited, the lower the ELA. 

We used the ELA method to process the input image, as shown below:

1. Save the original image and compress the input image to generate a new image according to the specified quality factor.
2. Calculate the absolute value of the difference between the two images pixel by pixel, and generate a difference set image.
3. According to the biggest pixel value of the difference set, we obtain the enhancement factor.
4. Adjust the brightness of the difference set image according to the enhancement factor, and generate the final enhancement ELA image.

**Figure 5:**
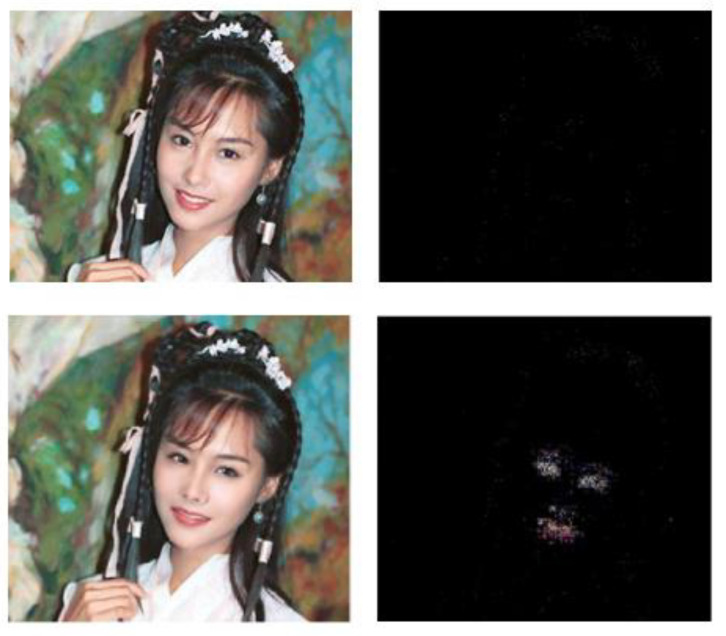
Samples of the original and tampered images and the error level analysis results.

The ELA processing effect is shown in Figure 5. The images on the first line are the original image and its ELA image. It can be seen that the compression ratio of the whole image remained the same. The images on the second line are the tampered image and its ELA image. It can be seen that the compression ratio between the tampered face as the foreground and the original image as the background were quite different.

As shown in Figure 5, the ELA method can be used to detect whether the image has been tampered with. However, the ELA method also has the following problems. First, it is only applicable to the compression distortion of lossy image compression such as the JPEG format and it cannot be used for the detection of lossless compression. All ELA tests were compared with JPEG lossy compression. Second, the ELA method can only roughly determine which areas of the image have been processed, which is more difficult to distinguish for low quality images.

### 3.3. CNN Architectures

Convolution neural network (CNN) is a kind of feedforward neural network with a depth structure and convolution computation. It is inspired by the human visual nervous system, and has two major characteristics: it can effectively reduce the dimension of a large amount of data to a small amount of data, and it can effectively retain the characteristics of the picture, in line with the principle of picture processing. CNN has achieved great success in the field of digital image processing such as object detection [19], face detection [20], face recognition [21], video classification [22], super resolution [23], and so on.

A typical CNN consists of three parts: the convolution layer, pooling layer, and full connection layer. Generally speaking, the convolution layer is responsible for extracting the local features from the input image; the pooling layer is used to greatly reduce the parameter order of magnitude (dimensionality reduction); and the full connection layer is similar to the part of the traditional neural network, which is used to output the desired results. From the point of view of signal processing, the convolution operation in the convolution layer is a filter (convolution kernel) to filter the frequency of the signal. The training of the CNN is to find the best filter (Filter) to make the filtered signal easier to classify. From the point of view of template matching, each convolution kernel can be regarded as a feature template, and the training is to obtain a suitable filter so that the specific mode can be highly activated to achieve the purpose of classification or detection. Unlike the convolution in the image, the convolution layer of the CNN can set multiple filters to obtain different feature maps. Furthermore, the value of each filter is not fixed, but variable and trainable.

**Figure 6:**
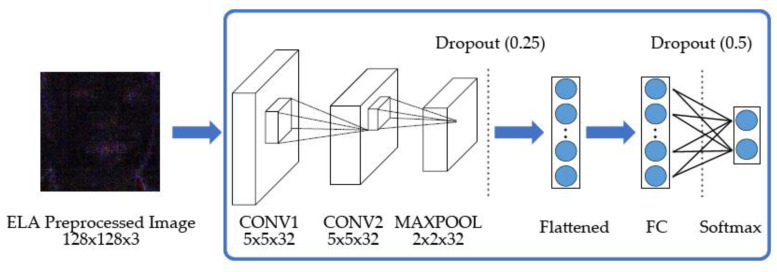
The trained CNN network architecture.

The CNN draws lessons from the working principle of the human visual system. The convolution neural network first obtains some low-level features by finding the edges or curves of the input image, and then aggregates these low-level features into more high-level ones through a series of convolution layers. As these high-level features are composed of multiple low-level features, the high-level features can cover more information of the original image. The CNN architecture used in our method is described in Figure 6.

For the details of the deep learning model, we used the following settings. The first CNN layer consisted of a convolutional layer with a kernel size of  5×5  and 32 filters. The second CNN layer consisted of a convolution layer with a kernel size of  5×5 , 32 filters, and a max-pooling layer with a kernel size of  2×2 . Both convolution layers use the Glorot uniform initializer kernel and the Relu activation function to create neurons on the convolution layer and perform selection so that they can receive useful signals from the input data. After that, add a dropout of 0.25 to the max-pooling layer to prevent over-fitting. The next layer is a fully connected layer with 256 neurons and Relu activation functions. After the fully connected layer, a dropout of 0.5 is added to prevent over-fitting.

The root mean square prop (Rmsprop) optimizer is one of the adaptive learning rate methods. The Rmsprop optimizer uses the same concept of the exponentially weighted average of the gradients like gradient descent with momentum, but the difference lies in the update of parameters. It limits the oscillations in the vertical direction, so that our algorithm can take a larger step in the horizontal direction and converge faster.

In Rmsprop, instead of using dW and db independently for each epoch, we took the exponentially weighted average of the square of dW and db.

(7) $$s_{dw}=\beta \times s_{dw}+\left(1-\beta \right)\times dW^{2}\;s_{db}=\beta \times s_{db}+\left(1-\beta \right)\times db^{2}$$

In the above formula, $s_{dw}$ and $s_{db}$ are the gradient momentum accumulated by the loss function in the first t−1 iteration, respectively. β is another hyperparameter and takes values from 0 to 1. It sets the weight between the average of previous values and the square of the current on to calculate the new weighted average.

After calculating the exponentially weighted averages, we update our parameters. The difference is that the Rmsprop algorithm calculates the differential square weighted average for the gradient. This method is beneficial to eliminate the direction with a large swing amplitude, and is used to modify the swing amplitude, so that the swing amplitude of each dimension is smaller. On the other hand, it makes the network function convergence faster.

(8) $$W=W-\alpha \times \frac{dW}{\sqrt{s_{dw}}+\varepsilon }\;b=b-\alpha \times \frac{db}{\sqrt{s_{db}}+\varepsilon }$$

wherein α is learning rate. In order to prevent the denominator from being zero, a very small value ε is used for smoothing and generally the value is 10^−8^. In the above formula, $s_{dw}$ is relatively small so that here we divide dW by a relatively small number whereas $s_{db}$ is relatively large, so that here we divide db with a relatively larger number to slow down the updates on a vertical dimension.

The output layer uses the softmax loss function, which is composed of the softmax classifier and the cross-entropy loss function. Softmax normalizes the output of classification prediction, and obtains the probability distribution of a sample point belonging to each category. For example, the probability of belonging to category *j* is:

(9) $$p\left(x_{j}\right)=soft\max(z_{j})=\frac{e^{z_{j}}}{\sum_{i=1}^{n}e^{z_{i}}}$$

The above formula is the softmax function. This result satisfies the standardization requirement of probability distribution: the output probability of all categories is not less than 0, and the sum of the output probabilities of all categories is equal to 1.

Kullback–Leibler (KL) divergence, which is also known as relative entropy, can be used to measure the difference between the two separate distributions p and q, and can be written as $D_{KL}(p∥q)$. In the context of machine learning, $D_{KL}(p∥q)$ is often called the information gain achieved if p is used instead of q.

(10) $$D_{KL}(p∥q)=\overset{n}{\underset{i=1}{\sum }}p\left(x_{i}\right)\log(\frac{p\left(x_{i}\right)}{q\left(x_{i}\right)})$$

where n is all the possibilities of the event. In machine learning, p is often used to represent the real distribution of the samples. For example, [1,0,0] indicates that the current samples belong to the first category and q is used to represent the distribution predicted by the model such as [0.7,0.2,0.1]. The smaller the value of $D_{KL}$, the closer the distribution of q and p.

From the perspective of information theory, the minimizing cross-entropy loss can be seen as minimizing the KL divergence of real distribution p and predicted probability distribution q. By deforming the above formula, we can get

(11) $$D_{KL}(p∥q)=-H\left(p\left(x\right)\right)+\left[-\overset{n}{\underset{i=1}{\sum }}p\left(x_{i}\right)\log(q\left(x_{i}\right))\right]$$

The former part of the equation happens to be the entropy of p, and the latter part of the equation is the cross-entropy:

(12) $$H\left(p,q\right)=-\overset{n}{\underset{i=1}{\sum }}p\left(x_{i}\right)\log(p\left(x_{i}\right))$$

In machine learning, we need to evaluate the gap between the label and predictions. Using KL divergence is only good, that is, $D_{KL}(y∥y\prime )$. As the former part of KL divergence −H(y) is constant, we only need to pay attention to cross-entropy in the optimization process. Therefore, in machine learning, especially in neural network classification problems, cross-entropy is used as the loss and evaluation model directly.

## 4. Experiments 

In this section, we first introduce the MUCT database for immediate use. Then, we preprocess the dataset and process it with the ELA method. Finally, we train and evaluate our model on it, and then make comparisons with other methods.

### 4.1. The Milborrow University of Cape Town Database

Deep learning mainly depends on tens of thousands of samples, especially in the generation and detection of face forgery images. It is based on the huge number of samples that DeepFakes can learn powerful image features through a convolution neural network. Through supervised training, they can learn more features that are imperceptible to human beings. On the other hand, DeepFakes need to use a large amount of manual training data and is thereby limited by the size of the existing dataset, the training time, and other factors. As a result, there are slight artificial traces between the face video generated by DeepFake and the real face. Therefore, an appropriate dataset is particularly important.

Although there are some datasets [24,25,26,27] for image tampering detection, they are inappropriate for large-scale facial tampering detection as there are insufficient tampering samples concentrated on facial areas. The Columbia Image Splicing dataset [24] and Institute of Automation, the Chinese Academy of Sciences (CASIA) [25,26] are large, but most of the tampered areas are not human faces. The DSI-1 dataset [27] focuses on facial tampering, but the sum of the tampered images is only 25. Therefore, it is difficult to train deep learning models on these datasets to detect facial tampering.

**Figure 7:**
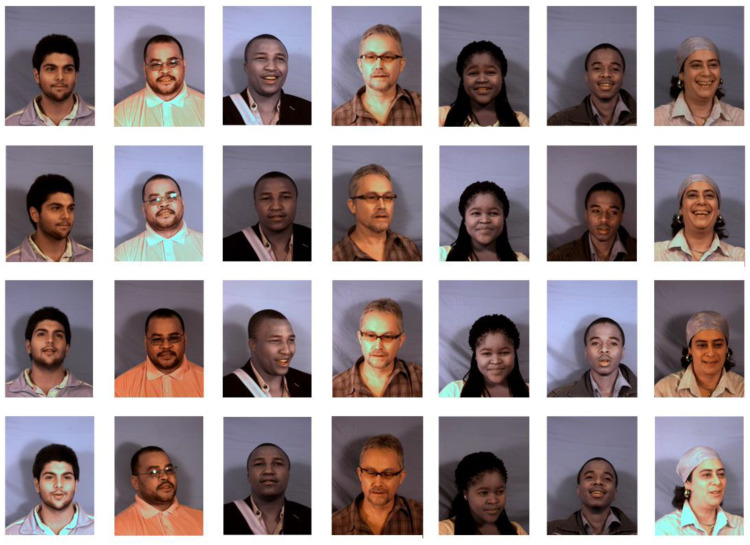
Samples from the MUCT database.

To do this, we used the Milborrow University of Cape Town (MUCT) database [28], which consists of 3755 facial images and 76 manual facial landmarks, as shown in Figure 7. Each compressed file in the data corresponds to a camera, providing more diverse lighting, age, and races than the currently available 2D face database. Since the facial images in the MUCT database are real, we need to preprocess it to generate our dataset with real and fake samples.

### 4.2. Dataset Preprocessing and ELA Processing

We used the 3755 “.jpg” format face images in the MUCT database as the examples, and the negative examples can be generated by simulating the DeepFake algorithm, as is shown in Figure 3, but it requires us to train and run DeepFake, which is a time-consuming and resource-demanding algorithm. Therefore, we used the method in Section 3.1 to generate negative examples dynamically and train them. Dynamic generation means that instead of generating all the negative examples in advance before the training process, we randomly selected half of the positive examples for each training batch and converted them to negative examples to make the training data more diverse. After the 128 × 128 ROI region images were generated in the previous step, we used the ELA method to process them and obtain their ELA images.

**Figure 8:**
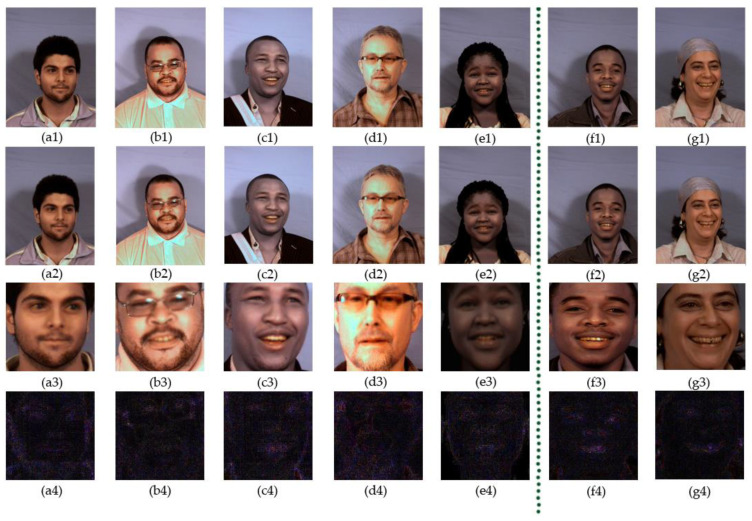
Samples of dataset preprocessing and the error level analysis processing: (**a**–**g**) are the samples of the MUCT dataset; (1–4) are the corresponding processing and ELA processing results of the original images.

Some samples of dataset preprocessing and ELA processing can be seen in Figure 8. The images on the first line are the original real images from the MUCT database. The images between a2 and e2 on the second line are the negative examples generated by imitating the DeepFake algorithm. Images e2 and f2 are the original positive examples. All the images on the second line were processed by image enhancement and other techniques, and retained only ROI areas. The images on the fourth line are the ELA images processed by the ELA method.

In the meantime, as can be seen from Figure 8, the original image areas are too large to contain much useless information, which has nothing to do with counterfeit traces. Instead, the retaining ROI areas save the most critical region, which is helpful to extract the most effective counterfeit features.

### 4.3. Train and Evaluate Model

The CNN model that we trained used these ELA images, rather than the original ones. It can be shown in Figure 6 as follows. Converting the original image to an ELA image is a method to improve the training efficiency of the CNN model. As the ELA image does not contain as much information as the original image, it can improve the efficiency. 

The feature generated by the ELA image focuses on the part of the original image where the error level is higher than the threshold value. In addition, the pixels in the ELA image are often quite different from the nearby pixels, and even the contrast is very obvious, so the image processed by ELA makes the training CNN model more effective. Therefore, we trained a CNN model to extract the counterfeit features of the ELA images, then detected whether the input image was forged or not. 

In the architecture that we used, only two convolution layers are required, because the ELA images generated in the previous steps can highlight the characteristics of the original image where the error level is higher than the threshold value. Therefore, it is easier to extract counterfeit features and determine whether the image is fake.

**Figure 9:**
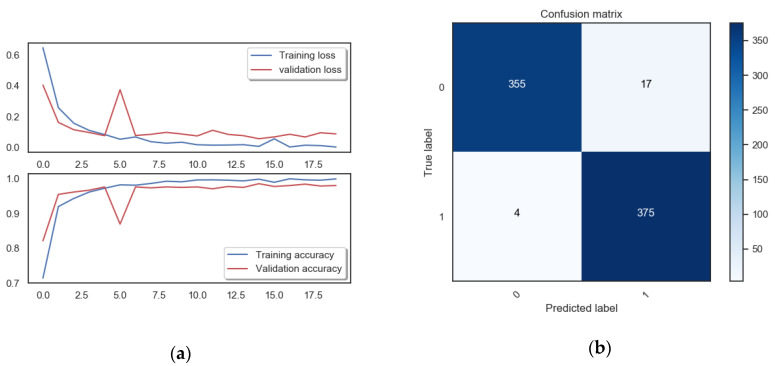
The experimental result images: (**a**) The image of the accuracy curve and the loss function curve. The horizontal axis is the number of training cycles, and the vertical axis represents the loss value and accuracy respectively. (**b**) The confusion matrix of the verification data.

We used the maximum accuracy for evaluation. The maximum accuracy of the results obtained by our proposed method was 97%. The image of the accuracy curve and the loss function curve can be seen in Figure 9a. The confusion matrix of the verification data is shown in Figure 9b.

As shown in Figure 9, our model achieved the best accuracy in the ninth cycle. From the first nine cycles later, we verified that the value of the loss function started to be flat and eventually began to increase, which is a sign of over-fitting. This is also a recognition method of ending training in advance during training, that is, when the verification accuracy value begins to decrease or the verification loss value starts to increase, the training will be stopped.

### 4.4. Comparison with Other Methods

We compared different models to prove the effectiveness of our method. We named the method [6] of training by directly using the CNN without ELA processing as a direct detection method. Its code is available from the public implementation on GitHub [29]. In this method, the images of positive and negative examples are directly input to the network model for training. Additionally, this method trains four CNN models: VGG16 [7], ResNet50, ResNet101, and ResNet152 [8].

The VGG16 network is named because it has 16 layers, that is, 13 convolution layers and three full connected layers. As VGG-Nets has good generalization performance, its pre-training model on the ImageNet dataset is widely used in many problems such as feature extraction. However, when the deep CNN network reaches a certain depth, its classification performance cannot be improved with the increase in the number of layers. In contrast, the network convergence becomes slower and the classification accuracy of the test dataset becomes worse. In order to overcome the problem where the learning efficiency becomes low and the accuracy cannot be effectively improved due to the deepening of the network (also known as network degradation), a deep residual network (ResNet) [8] was proposed. ResNet has different network layers, and the more commonly used ones are the 50-layer, 101-layer, and 152-layer. Nowadays, Resnet has replaced VGG as the basic feature extraction network in the field of computer vision such as face living detection [30].

The comparison results between our method and the direct detection methods are shown in Table 2. The area under curve (AUC) metric performance on VGG16, ResNet50, ResNet101, and ResNet152 reached 83.3%, 95.4%, 95.1%, and 93.8%. This table shows that our proposed method performed better than the direct detection methods. Additionally, the training efficiency of the CNN model could be significantly improved by using the ELA method.

As can be seen from Table 2, in terms of space complexity, the number of weight layers in the four models selected by the direct detection methods ranged from 16 to 152, with that of the parameters fluctuating between 6.03 × 10^7^ and 1.38 × 10^8^. In contrast, the number of layers and parameters in our method was three and 2.95 × 10^7^, respectively. In terms of time complexity, the model size in the four models selected by the direct detection methods varied from 98 MB to 736 MB, with the giga floating-point operations per second (GFLOPS) between 3.9 and 15.5. In contrast, in our method, this was 225 MB and 0.44 MB, respectively. In the practical experiment, the four models of this method needed to train more epochs in order to obtain satisfactory results, reaching 20 or 100 epochs. On the contrary, our method needed only nine epochs to reach the more satisfactory result.

The advantages of our model are as follows: the number of training periods required to achieve convergence is significantly reduced because the image features processed by ELA make the training more efficient, and accelerate the convergence of the CNN model. Furthermore, the accuracy of our classification results was relatively much higher. Coupled with the experiments showing that the training efficiency of the CNN model can be significantly improved by using the ELA method, this indicates that our method is feasible in simplicity and efficiency.

## 5. Conclusions

A marked improvement in the quality and efficiency in generating false faces could be seen evidently. In this paper, we studied a novel counterfeit feature extraction method based on deep learning, which could effectively distinguish fake facial images generated by deep learning.

We evaluated our method and proved its effectiveness in practice. This indicates that the features in the image processed by ELA can be successfully used to detect the authenticity of images. Furthermore, our experiments showed that the training efficiency of the CNN model can be significantly augmented through the ELA method. Specifically, without any loss in accuracy, the amount of computation can be significantly reduced (where the required floating-point computing power is reduced by more than 90%).

There are several directions that we will continue to improve our work. First, the method proposed in this paper is suitable for detecting image tampering under lossy compression, but it is not ideal for detecting tampering under low quality or lossless compression. Second, as the detection of image forgery and image composition is in a constant arms-race [31], the advanced technology behind DeepFake will continue to develop. Therefore, we will continue to evaluate and improve the robustness of our detection method.

## Figures and Tables

**Table 1:** Related work of deep learning in the field of image forensics (face-swap image detection).

Level Class	Combination with Forensics	Typical Examples
Level 1	——	four CNN models [6]
Level 2	——	MesoNet [9]
Level 3	abnormal physiological signal	breathing, pulse, eye blinking [11], head positions [13]
	Consistency of imaging equipment	Local noise residuals and camera characteristics [14]
	abnormal computer generated image	color difference [12]

**Table 2:** Comparison with four models.

Model	Space Complexity		Time Complexity		Practical Experiment	
	Weight Layers	Model Size (Parameters)	Model Size (MB)	GFLOPs (Forward Pass)	Training Epoch	Testing AUC
VGG16	16	1.38 × 10^8^	528 MB	15.5	100	83.3%
ResNet50	50	2.56 × 10^7^	98 MB	3.9	20	95.4%
ResNet101	101	4.46 × 10^7^	542 MB	7.6	20	95.1%
ResNet152	152	6.03 × 10^7^	736 MB	11.3	20	93.8%
Our method	3	2.95 × 10^7^	225 MB	0.44	9	97.6%
